# Nme Gene Family Evolutionary History Reveals Pre-Metazoan Origins and High Conservation between Humans and the Sea Anemone, *Nematostella vectensis*

**DOI:** 10.1371/journal.pone.0015506

**Published:** 2010-11-11

**Authors:** Thomas Desvignes, Pierre Pontarotti, Julien Bobe

**Affiliations:** 1 UMR 6632/IFR48, Université de Provence Aix Marseille 1/CNRS, F-13000, Marseille, France; 2 IFREMER, LALR, F-34250, Palavas les flots, France; 3 UMR 6632/IFR48, Université de Provence Aix Marseille 1/CNRS, F-13000, Marseille, France; American Museum of Natural History, United States of America

## Abstract

**Background:**

The Nme gene family is involved in multiple physiological and pathological processes such as cellular differentiation, development, metastatic dissemination, and cilia functions. Despite the known importance of Nme genes and their use as clinical markers of tumor aggressiveness, the associated cellular mechanisms remain poorly understood. Over the last 20 years, several non-vertebrate model species have been used to investigate Nme functions. However, the evolutionary history of the family remains poorly understood outside the vertebrate lineage. The aim of the study was thus to elucidate the evolutionary history of the Nme gene family in Metazoans.

**Methodology/Principal Findings:**

Using a total of 21 eukaryote species including 14 metazoans, the evolutionary history of Nme genes was reconstructed in the metazoan lineage. We demonstrated that the complexity of the Nme gene family, initially thought to be restricted to chordates, was also shared by the metazoan ancestor. We also provide evidence suggesting that the complexity of the family is mainly a eukaryotic innovation, with the exception of Nme8 that is likely to be a choanoflagellate/metazoan innovation. Highly conserved gene structure, genomic linkage, and protein domains were identified among metazoans, some features being also conserved in eukaryotes. When considering the entire Nme family, the starlet sea anemone is the studied metazoan species exhibiting the most conserved gene and protein sequence features with humans. In addition, we were able to show that most of the proteins known to interact with human NME proteins were also found in starlet sea anemone.

**Conclusion/Significance:**

Together, our observations further support the association of Nme genes with key cellular functions that have been conserved throughout metazoan evolution. Future investigations of evolutionarily conserved Nme gene functions using the starlet sea anemone could shed new light on a wide variety of key developmental and cellular processes.

## Introduction

The Nme family, initially called NDPK or Nm23, was named after the identification of a novel gene associated with low metastatic potential [1]. In humans, NME genes are involved in a wide variety of physiological or pathological cellular processes including development, metastatic potential, ciliary functions, and cell differentiation and proliferation at various tissular and subcellular localization (see [2] for recent review). Despite their critical role in key developmental and pathological processes, the molecular functions of Nme genes remain poorly documented [3]. In vertebrates, Nme genes can be separated in 2 groups – group I and group II – based on their evolutionary history and protein domains [4]. Nme genes of the group I (Nme1-4) originate from a unique gene of the chordate ancestor while Nme genes of the group II (Nme 5-8) are present throughout chordate evolution [4]. Nme-related genes have been sporadically reported in Archaea [5], Eubacteria [6], [7], and in several eukaryotic lineages including fungi [8], plants [9], and bilaterians [10], [11]. However, the evolutionary history of the family that has led to a repertoire of 5 Nme genes in the chordate ancestor remains poorly understood. Indeed, the complexity of the Nme gene repertoire outside the chordate lineage was previously uncharacterized and existing literature suggested that the complexity of the Nme gene family was much more limited in non-chordate species. In *Dictyostelium discoideum*, two Nme-related proteins, named NdkC-2 and NdkM, and expressed in the cytosol and in the mitochondria, respectively, were used for biochemical and structural studies [12]–[14]. In *Drosophila melanogaster,* only one Nme-related gene, named *awd*, had been reported and intensively studied for its role in aberrant development [10]. In *Caenorhabditis elegans*, one Nme-related gene had been shown to be associated with severe developmental defects [15]. As recently stressed, the use of model species to decipher Nme gene functions is extremely beneficial and needs to be further supported [16]. However, a better understanding of the evolutionary links between the Nme genes that are found in non-vertebrate model species and their mammalian counterparts is required to allow this comparative biology approach. In order to gain insight into putatively conserved key functions of Nme genes that would have been retained throughout evolution, the aim of the present study was thus to characterize gene family complexity and protein features among metazoans. We were able to show that the complexity of the Nme family predates the metazoan radiation. We also provided evidence supporting the association of Nme genes with key cellular functions that have been conserved throughout metazoan evolution.

## Results/Discussion

### *Nme* gene family complexity predates Metazoan radiation

**Figure 1:**
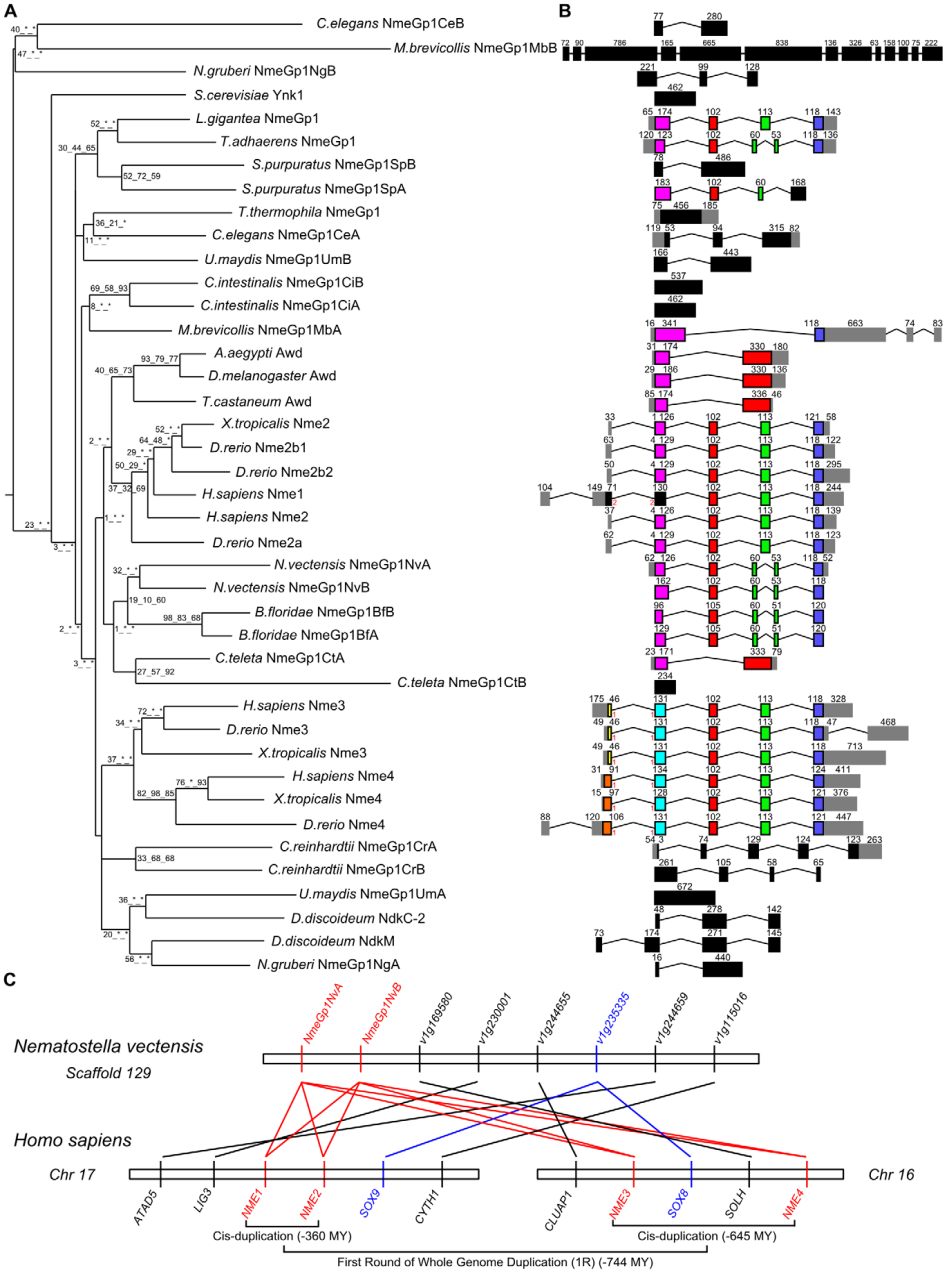
Phylogenetic tree and exon-intron structure of Group I Nme in Eukaryotes and synteny conservation between human and starlet sea anemone Group I Nme. A. The phylogenetic tree was constructed from a single multiple sequence alignment. Bootstrap values for neighbor joining, maximum parsimony, and maximum likelihood methods, respectively, are indicated for each node. Asterisks (*) indicate that the node was not recovered by the corresponding phylogenetic method. The consensus midpoint-rooted tree was calculated using the FIGENIX automated phylogenomic annotation pipeline [41]. For each sequence, species and corresponding name are shown. B. Corresponding exon/intron structures of genes coding for the proteins used for the phylogenetic tree estimation. Exon/intron structure was obtained through Ensembl, NCBI or JGI databases. When exon boundaries correspond to identical amino acid positions, the exons are displayed in color. Otherwise, exons are displayed in black. Non-coding exons are shown in grey. Exon size (in nucleotides) is indicated above each box. C. Synteny analysis between *Nematostella vectensis NmeGp1* genes and human group I *NME* genes. *Nematostella vectensis* gene names are NCBI Entrez gene symbols. For clarity reasons “NEMVEDRAFT_” was removed from all gene symbols. Duplication time estimations are from TimeTree [46].

Using available sequenced genomes of 21 eukaryote species ranging from amoebozoans to humans, we were able to reconstruct, in opisthokonts – the metazoan/choanoflagellate/fungi phylum – the evolutionary history of the Nme genes that had previously been identified in the chordate ancestor [4]. We were able to show that the metazoan and chordate ancestors share a similar Nme gene repertoire. A similar repertoire was also found in *Monosiga brevicollis*, a choanoflagellate, but not in fungi (Figures 1–5).

**Figure 2 pone-0015506-g002:**
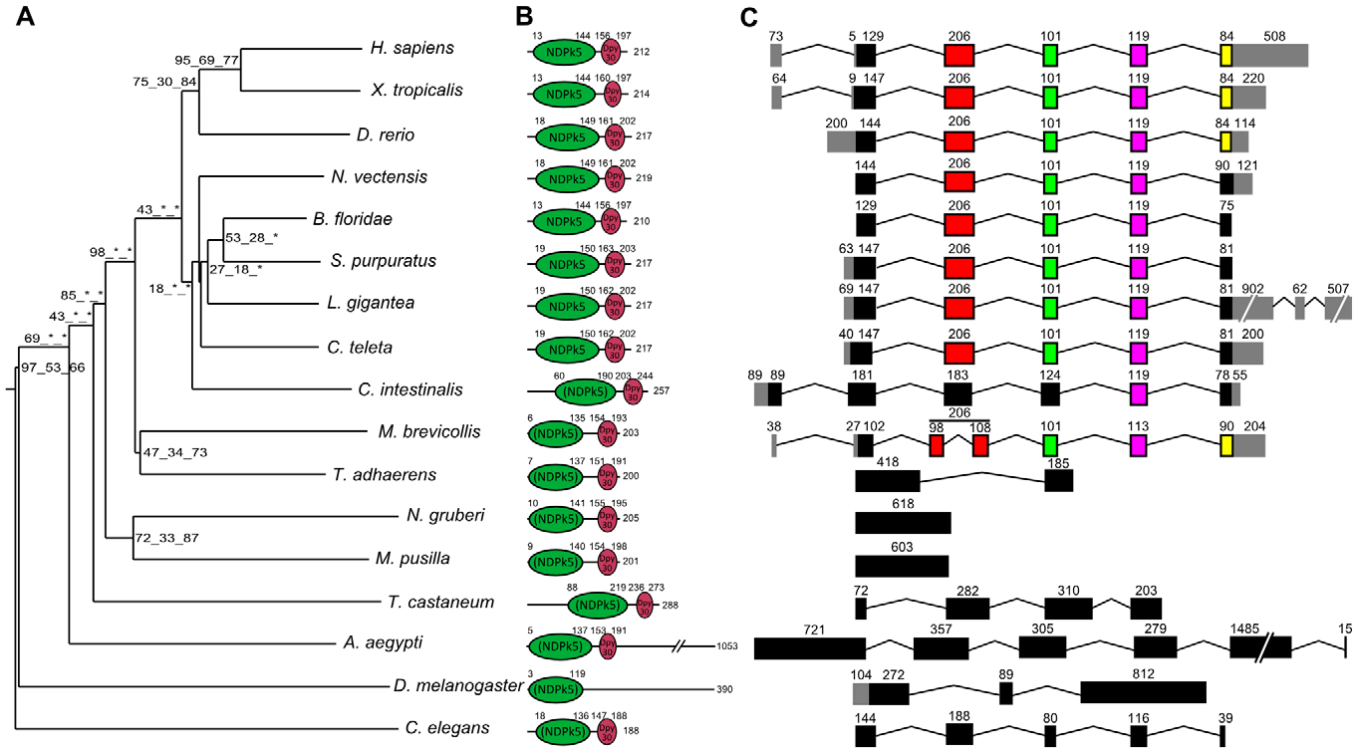
Phylogenetic tree, protein domains, and exon-intron structure of Nme5 in Eukaryotes. A. The phylogenetic midpoint-rooted tree was constructed as described in Figure 1A. B. Corresponding protein domain structure of Nme proteins. Protein domain information was obtained using Genbank Conserved Domain Database [40]. Parentheses indicate that the domain type is the best hit given by NCBI CDD but is not a specific hit according to CDD default parameters. C. Exon/intron gene structure was obtained as described in Figure 1B.

**Figure 3 pone-0015506-g003:**
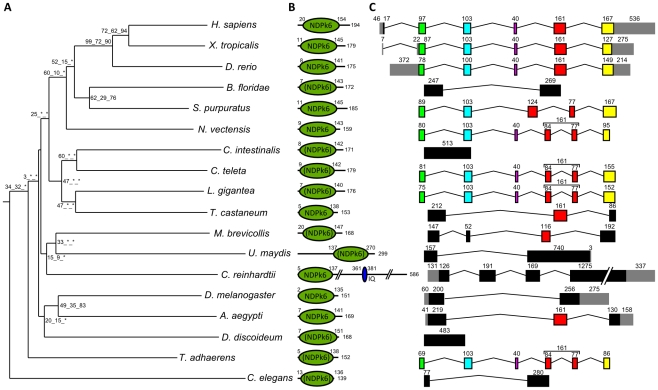
Phylogenetic tree, protein domains, and exon-intron structure of Nme6 in Eukaryotes. A. The phylogenetic midpoint-rooted tree was constructed as described in Figure 1A. B. Corresponding protein domain structure was obtained as described in Figure 2A. C. Exon/intron gene structure was obtained as described in Figure 1B.

**Figure 4 pone-0015506-g004:**
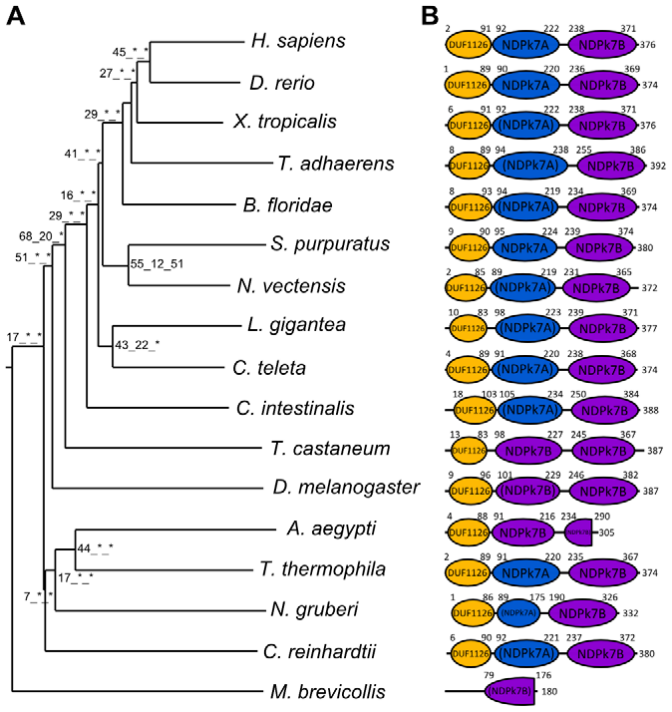
Phylogenetic tree and protein domains of Nme7 in Eukaryotes. A. The phylogenetic midpoint-rooted tree was constructed as described in Figure 1A. B. Corresponding protein domain structure was obtained as described in Figure 2A.

**Figure 5:**
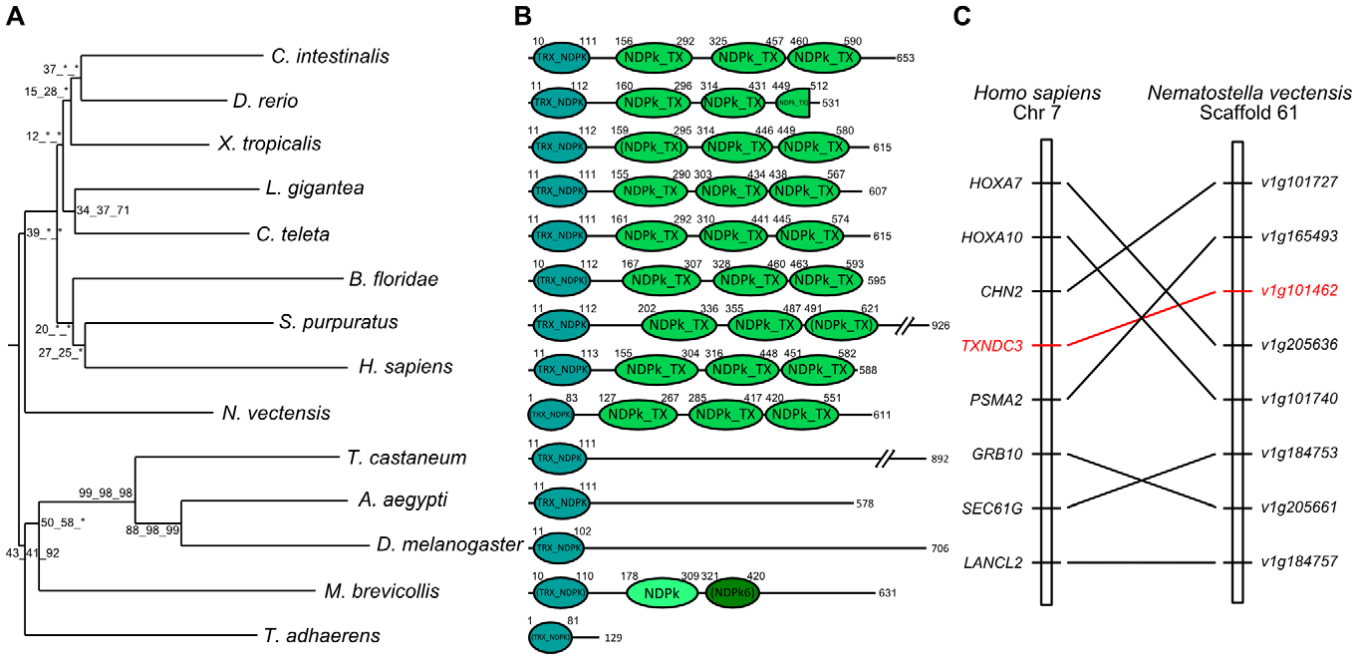
Phylogenetic tree and protein domains of Nme8 in Metazoans. A. The phylogenetic midpoint-rooted tree was constructed as described in Figure 1A. B. Corresponding protein domain structure was obtained as described in Figure 2A. C. Synteny analysis between *Nematostella vectensis Nme8* genes and human *TXNDC3* (*NME8*) genes. *Nematostella vectensis* gene names correspond to the NCBI Entrez Gene identification. For clarity reasons the unchanged part “NEMVEDRAFT_” was removed.

All non-vertebrate metazoans species displayed 2 Nme genes of the group I with the exception of *Trichoplax adhaerens*, *Drosophila melanogaster, Aedes aegypti, Tribolium castaneum,* and *Lottia gigantea*, in which a single gene could be identified (Figure 1A–B). In all studied non-metazoan eukaryotes, two Nme genes of the group I could also be identified with the exception of *Saccharomyces cerevisiae* and *Tetrahymena thermophila*. In starlet sea anemone *Nematostella vectensis*, *Caenorhabditis elegans* and *Branchiostoma floridae*, the two genes are located tandemly and thus originate from a *cis*-duplication of an ancestral gene (Table 1). In addition, a surprisingly well conserved gene synteny of group I Nme sequences was found between sea anemone and humans (Figure 1C). The location of the *Sox8/9* ancestor gene in the vicinity of Nme genes in the sea anemone and the location of *SOX8* and *SOX9* on human chromosomes 17 and 16, respectively, are consistent with the first round of whole genome duplication (1R) that gave rise to *Nme2* and *Nme3/4* in vertebrates [4]. In *Ciona intestinalis*, we have obtained evidence suggesting a duplication of a large portion of genomic DNA resulting in duplicated genes on two different chromosomes (Table 1, Figure S1). Two duplicated Nme genes of the group I originating from a common group I ancestral gene were found on chromosomes 2q and 8q (Figure S1). In all studied non-metazoan eukaryotes, *Capitela teleta*, and *Strongylocentrotus purpuratus*, two Nme genes of the group I can be found on different scaffolds. Because of the limited size of these scaffolds and the level of assembly of corresponding draft genomes, it is not currently possible to speculate on the nature of the duplication event that has led to 2 genes. In *Dictyostelium discoideum*, 2 Nme genes of the group I could be identified on different chromosomes. The topology of the phylogenetic tree displaying – for *Chlamydomonas reinhardtii*, *Nematostella vectensis*, *Capitela teleta*, *Strongylocentrotus purpuratus*, *Branchiostoma floridae*, and *Ciona intestinalis* – the group I Nme sequences more closely related within a species than between species strongly suggests that corresponding duplication events are independent and lineage-specific. It is noteworthy that for the above species, duplicated sequences remained closely related, whereas in other species, *Naegleria gruberi*, *Ustilago maydis*, *M. brevicollis*, and *C. elegans* one sequence is highly divergent in comparison to the other one. Altogether, this strongly suggests independent and lineage specific gene duplications. It was previously shown that a single Nme gene of the group I was present in the vertebrate ancestor [4]. This ancestor gene subsequently duplicated differently in the different vertebrates lineages and resulted in 2 to 5 genes, depending on the species [4]. Together, our data indicate that a single Nme gene of the group I was present in the opisthokont ancestor. Our observations also suggest that a single Nme gene of the group I was present in the eukaryote ancestor. Understanding the selective factors that have led to multiple independent duplications events in the *Nme* family and the role of these proteins in non-vertebrate species would provide major insights into the evolution and functions of the family.

**Table 1:** GroupI Nme proteins: names and symbols by species, accession numbers and corresponding chromosomal location.

	Species	Name	GenBank Acc. no.	Ensembl or JGI Acc. no.	Localisation	Position
	***N. gruberi***	NmeGp1NgA	XP_002672581	72250	Scaffold_52	70,213–70,711
	***N. gruberi***	NmeGp1NgB	XP_002678151	60551	Scaffold_19	18,232–18,965
	***T. thermophila***	NmeGp1	XP_001018488		Scaffold_8254597	472,318–473,033
	***D. discoideum***	NdkC-2	XP_644519	DDB0238334	Chr 2	3,427,961–3,428,694
	***D. discoideum***	NdkM	XP_641417	DDB0214817	Chr 3	2,740,248–2,741,470
	***C. reinhardtii***	NmeGp1CrA	XP_001698246	58944	Chr 16	365,385–367,897
	***C. reinhardtii***	NmeGp1CrB	XP_001702884	292075	Scaffold_26	140,833–142,142
	***U. maydis***	NmeGp1UmA	XP_759114	2967	Contig_1_101	162,394–163,065
	***U. maydis***	NmeGp1UmB	XP_758923	2776	Contig_1_94	34,001–34,701
	***S. cerevisiae***	Ynk1	NP_012856	YKL067W	Chromosome XI	314,456–314,917
	***M. brevicollis***	NmeGp1MbA	XP_001742597	19073	Scaffold_2	878,808–880,474
	***M. brevicollis***	NmeGp1MbB	XP_001749207	28652	Scaffold_28	66,018–71,920
	***T. adhaerens***	NmeGp1	XP_002115688	49508	Scaffold_11	2,334,568–2,336,179
	***N. vectensis***	NmeGp1NvA	XP_001630018	169568	Scaffold_129	58,094–60,224
	***N. vectensis***	NmeGp1NvB	XP_001630017	115087	Scaffold_129	51,034–52,881
	***C. elegans***	NmeGp1CeA	NP_492761	F25H2.5.1	Chr I	10,553,902–10,553,101
	***C. elegans***	NmeGp1CeB	NP_492819	F55A3.6	Chr I	10,784,780–10,783,989
	***T. castaneum***	Awd	XP_967503		Linkage Group 3	32,141,388–32,142,070
***Group1***	***D. melanogaster***	Awd	NP_476761	FBpp0085223	Chr 3R	27,570,894–27,571,706
***Nme***	***A. aegypti***	Awd	XP_001662512	AAEL012359-PA	SuperContig1.684	87,333–88,186
	***L. gigantea***	NmeGp1		205662	Scaffold_3	3,942,249–3,946,053
	***C. teleta***	NmeGp1CtA		21725	Scaffold_758	80,222–84,888
	***C. teleta***	NmeGp1CtB		116635	Scaffold_527	188,815–189,048
	***S. purpuratus***	NmeGp1SpA	XP_799145		Scaffold71291	17,770–21,754
	***S. purpuratus***	NmeGp1SpB	XP_785384		Scaffold42444	298,840–301,211
	***C. intestinalis***	NmeGp1CiA	XP_002123476	ENSCINP00000011619	Chr 8q	5,908,347–5,908,808
	***C. intestinalis***	NmeGp1CiB	XP_002121438	ENSCINP00000002194	Chr 2q	7,888,026–7,888,562
	***B. floridae***	NmeGp1BfA	XP_002598285	57540	Scaffold_24	2,074,549–2,076,844
	***B. floridae***	NmeGp1BfB	XP_002598283	69640	Scaffold_24	2,064,578–2,066,435
	***H. sapiens***	NME1	NP_937818	ENSP00000337060	Chr 17	49,230,937–49,239,422
	***H. sapiens***	NME2	NP_001018149	ENSP00000376888	Chr 17	49,243,639–49,249,108
	***X. tropicalis***	Nme2	NP_001005140	ENSXETP00000024764	Scaffold_673	77,640–81,451
***Nme2***	***D. rerio***	Nme2a	NP_956264	ENSDARP00000064338	Scaffold Zv8_scaffold3117	528,950–537,135
	***D. rerio***	Nme2b1	NP_571001	ENSDARP00000099319	Chr 19	48,501,402–48,504,320
	***D. rerio***	Nme2b2	NP_571002	ENSDARP00000098065	Chr 19	48,507,488–48,510,647
	***H. sapiens***	NME3	NP_002504	ENSP00000219302	Chr 16	1,820,321–1,821,710
***Nme3***	***X. tropicalis***	Nme3	NP_001005115	ENSXETP00000022770	Scaffold_27	933,363–937,018
	***D. rerio***	Nme3	NP_571003	ENSDARP00000075112	Chr 3	11,995,596–12,045,134
	***H. sapiens***	NME4	NP_005000	ENSP00000219479	Chr 16	447,209–450,759
***Nme4***	***X. tropicalis***	Nme4	NP_001039239	ENSXETP00000022726	Scaffold_27	1,305,654–1,312,714
	***D. rerio***	Nme4	NP_957489	ENSDARP00000103207	Chr 3	10,909,410–10,922,804

Protein names were retrieved from Ensembl and NCBI or proposed according to the evolutionary history of the genes. Chromosomal/genomic location was obtained using Ensembl genome browser, JGI databases, or NCBI Entrez Gene when not available on Ensembl or JGI.

All studied metazoan species displayed a full set of group II Nme genes with the exception of the 4 ecdysozoan species and *T. adhaerens* (Figures 2–5). The 2 other group II Nme genes, *Nme9* and *Nme10*, that have been shown to be eutherian and vertebrate innovations, respectively [4], will not be discussed here. No *Nme7* homolog could be identified in the *C. elegans* genome thus indicating a possible gene loss after the nematode radiation. In *M. brevicollis* the Nme7 protein is structurally highly divergent as shown but the topology of the phylogenetic tree (Figure 4A) and displays a unique and incomplete domain (Figure 4B). In insects, Nme7 proteins also displayed specific domain structure (Figure 4B) resulting in a divergent position of the corresponding group in the phylogenetic tree (Figure 4A). As for all phylogenetic analyses reported here, the tree topology remained unchanged whether we used the full length protein sequence or only domains for the phylogenetic reconstruction. Similarly, very divergent Nme8-related sequences were identified in insects and *T. adhaerens* (Figure 5). In these 4 species, the Nme8-related proteins have lost the 3 NDPK_TX domains that are found in all other metazoan species, including the starlet sea anemone (Figure 5). In *M. brevicollis*, the Nme8 protein does not display typical Nme8 NDPK_TX domains but 2 different domains of the NDPk superfamily. It is also noteworthy that the exon/intron structure corresponding to the Thioredoxin TRX_NDPK domain is very well conserved among all studied choanoflagellate and metazoan species. In contrast, the exon/intron structure corresponding to the NDPK domains is highly divergent in insects, placozoans and choanoflagellates (Figure S2). Together, our data demonstrate that *Nme5*, *Nme6*, *Nme7*, and *Nme8* genes were already present in the genome of the common ancestor of choanoflagellates and metazoans. In non-choanoflagellate/metazoan species, all Nme proteins of the group II were found in low branching eukaryotic lineages such as heteroloboseans, green plants, amoebozoans, alveolates, and fungi (Figures 2–4, Table 2) with the exception of Nme8. In contrast, we failed to identify Nme genes of the group II outside the eukaryotic lineage. Together, our results strongly suggest that *Nme5*, *Nme6*, and *Nme7* emerged around Eukaryote radiation. Interestingly, none of the studied non-choanoflagellate/metazoan species display all 3 proteins suggesting lineage specific loss of *Nme 5*, *Nme6*, and *Nme7* genes. The Nme8 protein typically displays 1 Thioredoxin (TRX) domain followed by 2 or 3 complete NDPk domains. In non-choanoflagellates/metazoan species investigated, very few sequences displaying one thioredoxin domain could be identified but were never associated with an NDPk domain. We thus hypothesize that Thioredoxin domains already existed in the opisthokont ancestor and that Nme8 emerged in the choanoflagellate/metazoan ancestor by domain shuffling of 2 or 3 NDPk domains. This would, however, require further analysis.

**Table 2:** GroupII Nme proteins: names and symbols by species, accession numbers and corresponding chromosomal location.

	Species	Name	GenBank Acc. no.	Ensembl or JGI Acc. no.	Localisation	Position
***Nme5***	***N. gruberi***	Nme5	XP_002682323	29950	Scaffold_3	679,761–680,378
	***M. pusilla***	Nme5	XP_003056664	15249	Scaffold_3	169,341–169,943
	***M. brevicollis***	Nme5	XP_001749876	38924	Scaffold_34	201,106–202,764
	***T. adhaerens***	Nme5	XP_002112439	50304	Scaffold_5	3,451,699–3,452,497
	***N. vectensis***	Nme5	XP_001631264	244029	Scaffold_105	447,049–449,367
	***C. elegans***	Nme5	NP_501212	R05G6.5	Chr IV	7,508,567–7,509,459
	***T. castaneum***	Nme5	XP_001811234		Linkage Group 2	13,741,320–13,742,593
	***D. melanogaster***	Nme5	NP_651833	FBpp0085077	Chr 3R	26,707,514–26,709,225
	***A. aegypti***	Nme5	XP_001662993	AAEL003030-PA	SuperContig1.75	2,400,537–2,515,468
	***L. gigantea***	Nme5		226200	Scaffold_168	228,601–233,346
	***C. teleta***	Nme5		177417	Scaffold_116	97,523–98,941
	***S. purpuratus***	Nme5	XP_790390		Scaffold25557	320,279–323,900
	***C. intestinalis***	Nme5	NP_001154961	ENSCINP00000008954	Chr 7q	1,031,987–1,036,756
	***B. floridae***	Nme5	XP_002596479	61845	Scaffold_430	334,501–336,787
	***H. sapiens***	NME5	NP_003542	ENSP00000265191	Chr 5	137,450,866–137,475,104
	***X. tropicalis***	Nme5	NP_001072619	ENSXETP00000008322	Scaffold_65	2,613–8,494
	***D. rerio***	Nme5	NP_001002516	ENSDARP00000060997	Chr 14	6,455,589–6,462,855
***Nme6***	***D. discoideum***	Nme6	XP_629447	DDB0191701	Chr 6	2,282,811–2,283,293
	***C. reinhardtii***	Nme6	XP_001698136	139197	Chr 16	825,573–828,346
	***U. maydis***	Nme6	XP_760135	3988	Contig_1_139	15,223–16,271
	***M. brevicollis***	Nme6	XP_001744091	15435	Scaffold_5	413,751–414,530
	***T. adhaerens***	Nme6	XP_002114036	58087	Scaffold_7	2,675,108–2,676,755
	***N. vectensis***	Nme6	XP_001622626	140474	Scaffold_525	60,519–64,497
	***C. elegans***	Nme6	NP_001021779	Y48G8AL.15	Chr I	1,254,771–1,258,597
	***D. melanogaster***	Nme6	NP_572965	FBpp0073750	Chr X	14,477,710–14,478,649
	***T. castaneum***	Nme6	XP_972639		Linkage Group 8	6,083,034–6,083,586
	***A. aegypti***	Nme6	XP_001648448	AAEL004107-PA	SuperContig1.107	2,016,668–2,033,334
	***L. gigantea***	Nme6		112601	Scaffold_15	3,040,725–3,042,846
	***C. teleta***	Nme6		91319	Scaffold_32	665,868–668,578
	***S. purpuratus***	Nme6	XP_001200902		Scaffold35436	11,834–18,064
	***C. intestinalis***	Nme6	XP_002129729	ENSCINP00000027945	Scaffold_1779	5,509–6,021
	***B. floridae***	Nme6	XP_002589414	279926	Scaffold_243	2,320,842–2,322,329
	***H. sapiens***	NME6	NP_005784	ENSP00000416658	Chr 3	48,334,754–48,342,848
	***X. tropicalis***	Nme6	NP_001123709	ENSXETP00000034257	Scaffold_857	287,305–293,502
	***D. rerio***	Nme6	NP_571672	ENSDARP00000094574	Chr 20	18,803,906–18,812,975
***Nme7***	***N. gruberi***	Nme7	XP_002677897	33146	Scaffold_20	272,036–273,256
	***C. reinhardtii***	Nme7	XP_001702841	180221	Chr 12	8,939,035–8,941,564
	***T. thermophila***	Nme7	XP_001015884		Scaffold_8254649	122,957–124,735
	***M. brevicollis***	Nme7	XP_001749106	11267	Scaffold_27	519,462–520,004
	***T. adhaerens***	Nme7	XP_002108466	51403	Scaffold_1	1,598,731–1,601,855
	***N. vectensis***	Nme7	XP_001626602	125694	Scaffold_222	17,379–24,092
	***T. castaneum***	Nme7	XP_974333		Linkage Group 7	18,619,008–18,620,171
	***D. melanogaster***	Nme7	NP_649926	FBpp0081561	Chr 3R	5,505,663–5,507,224
	***A. aegypti***	Nme7	XP_001661412	AAEL011098-PA	SuperContig1.541	206,988–220,408
	***L. gigantea***	Nme7		187020	Scaffold_18	1,179,880–1,185,739
	***C. teleta***	Nme7		160391	Scaffold_422	28,192–33,650
	***S. purpuratus***	Nme7	XP_795051		Scaffold9766	108,673–117,014
	***C. intestinalis***	Nme7	NP_001155162	ENSCINP00000025129	Chr 1p	3,423,461–3,424,231
	***B. floridae***	Nme7	XP_002588622	287848	Scaffold_254	342,459–356,708
	***H. sapiens***	NME7	NP_037462	ENSP00000356785	Chr 1	169,101,769–169,337,205
	***X. tropicalis***	Nme7	NP_988903	ENSXETP00000005150	Scaffold_169	1,646,165–1,680,298
	***D. rerio***	Nme7	NP_571004	ENSDARP00000073091	Chr 6	31,135,867–31,196,533
***Nme8***	***M. brevicollis***	Nme8	XP_001746342	32671	Scaffold_12	606,463–609,773
	***T. adhaerens***	Nme8	XP_002110931	22954	Scaffold_3	3,392,460–3,393,060
	***N. vectensis***	Nme8	XP_001634297	101462	Scaffold_61	602,301–615,863
	***T. castaneum***	TRX-Nme8	XP_972627		Linkage Group 5	12,145,420–12,148,468
	***D. melanogaster***	TRX-Nme8	NP_572772	FBpp0073425	Chr X	11,884,151–11,886,801
	***A. aegypti***	TRX-Nme8	XP_001652618	AAEL007253-PA	SuperContig1.245	180,440–198,050
	***L. gigantea***	Nme8		107502	Scaffold_7	645,416–655,143
	***C. teleta***	Nme8		96991	Scaffold_940	8,079–11,527
	***S. purpuratus***	Nme8	XP_001181827		Scaffold66657	21,472–41,768
	***C. intestinalis***	Nme8	NP_001027618	ENSCINP00000013583	Chr 9q	3,694,058–3,703,935
	***B. floridae***	Nme8	XP_002597926	280761	Scaffold_152	66,164–77,826
	***H. sapiens***	NME8	NP_057700	ENSP00000199447	Chr 7	37,888,199–37,940,003
	***X. tropicalis***	Nme8	NP_001121456	ENSXETP00000002355	Scaffold_664	444,670–467,036
	***D. rerio***	Nme8	NP_001082944	ENSDARP00000103107	Chr9	51,493,472–51,569,898

Protein names were retrieved from Ensembl and NCBI or proposed according to the evolutionary history of the genes. Chromosomal/genomic location was obtained using Ensembl genome browser, JGI databases, or NCBI Entrez Gene when not available on Ensembl or JGI.

**Figure 6:**
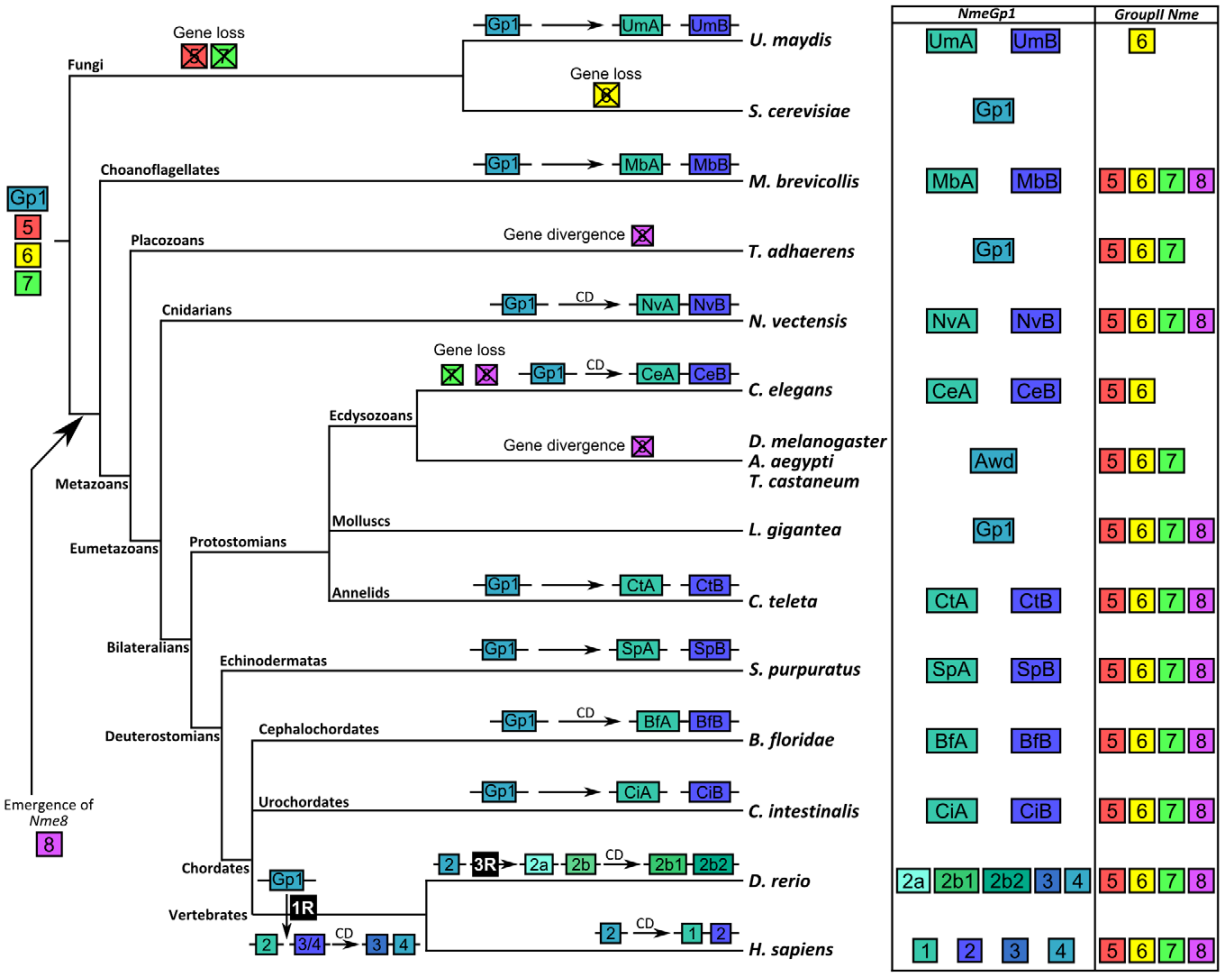
Schematic depiction of Nme genes evolution in Opisthokonts. The gene repertoire is shown at the root of the tree for the opisthokont ancestor. For all lineages, duplication events are shown and *cis*-duplications (CD) indicated. For the vertebrate lineage, 1R and 3R whole genome duplications events are shown and duplication events of group I Nme genes redrawn from [4]. Nme 9 and Nme10 that have been shown to be vertebrate innovations [4] are not displayed here for clarity reasons. The gene repertoire is given for all studied species. For clarity reasons, the complete name of the gene is not given and only the gene number is given and the color kept consistent with the corresponding gene in the opisthokont ancestor.

Here, we have shown that the Nme gene repertoire of the metazoan ancestor was similar to that of the vertebrate ancestor (Figure 6). In agreement with prior studies reporting major gene losses and genomic rearrangement experienced by ecdysozoans [17]–[20] we show that Nme genes have highly diverged in this phylogenetic group, resulting in the loss of either functional NDPK domains or entire genes. Furthermore, we demonstrate that the complexity of the family predates metazoan radiation. We also provide evidence suggesting that the complexity of the family is mainly a eukaryotic innovation, with the exception of *Nme8* that is likely to be a choanoflagellate/metazoan innovation. This unexpectedly ancient complexity of the eukaryotic Nme gene family is in striking contrast with the existing hypothesis associating the emergence of Nme family complexity with the differentiation of Bilateralian [21] lineage. It should be stressed that this burst of complexity in the Nme family is much more ancient than what has been reported for many genes in which the expansion of the family is thought to have occurred around the metazoan radiation [18], [19], [22], [23]. This would be in favor of the participation of Nme genes in ancestral functions and would also be consistent with their known involvement in key biological processes such as cell proliferation and development.

### Highly conserved *Nme* sequence features exist among Metazoans and Eukaryotes

The topology of the phylogenetic tree (Figure 1A) obtained using group I Nme proteins does not match the topology of the tree of life. Indeed, *N. vectensis* sequences appear, along with *B. floridae* and *C. teleta* sequences, as a sister group of the vertebrate and insect sequences, in contrast to *T. adhaerens*, *S. purpuratus*, and *C. intestinalis* sequences that appear much more divergent. The topology of the tree is, however, consistent with the genomic structure of group I Nme genes reported on Figure 1B. A highly conserved exon/intron structure is observed between *T. adhaerens*, *N. vectensis*, *L. gigantea*, and vertebrates, while ecdysozoans, *C. teleta*, *C. intestinalis,* and *S. purpuratus* exhibit a totally different genomic organization that reflects the high gene divergence observed in these species. Interestingly, the *D. discoideum* mitochondrial NdkM shows gene and protein features that are similar (Figure 1B) to its cytosolic paralog NdkC-2, but displays a longer N-terminus sequence containing a mitochondrial assignation signal. In vertebrates, the Nme4 protein also displays a mitochondrial assignation signal in N-terminus sequence and was shown to be a gnathostome innovation [4]. In contrast, no mitochondrial assignation signal is found in any other Nme protein. This feature is thus likely to be a functional convergence between amoebozoan NdkM and vertebrate Nme4.

As indicated above, orthologs of Human *Nme5*, *Nme6*, *Nme7*, and *Nme8* are found in metazoans (Figure S3). The topology of the phylogenetic tree (Figure 2A) obtained using Nme5 proteins showed that ecdysozoan sequences are highly divergent. Ecdysozoan Nme5 proteins appear to be even more divergent than the early diverging eukaryotes *N. gruberi* and *M. pusilla*. Similarly to group I, gene exon/intron structure and protein domains of *N. vectensis*, *B. floridae*, *S. purpuratus*, *C. teleta*, and *L. gigantea* Nme 5 proteins (Figures 2B and 2C) were highly similar to their human counterpart, while ecdysozoan sequences exhibited different protein length and/or domains. In agreement with these observations was the remarkably conserved exon/intron structure observed between humans and sea anemone *Nme5* genomic sequences (Figure 2C). Similar observations on tree topology, protein domains, and genomic structure were made for Nme6 (Figure 3). For Nme7, no ortholog could be identified in *C. elegans*, while very divergent Nme7 sequences were identified in non-metazoan eukaryote species, *M. brevicollis*, and insects as shown by the topology of the phylogenetic tree (Figure 4A). In contrast to all other studied species, no NDPk7A domain was found (Figure 4B) in *M. brevicollis* and insects sequences whereas they could be identified in several non-opisthokont eukaryote species, thus suggesting a high divergence of ecdysozoan genes within the metazoan lineage. In addition, an incomplete NDPk7B domain was found in *A. aegypti* (Figure 4B). It should be stressed that, in contrast to insects, the Nme7 sequences of *T. adhaerens*, *N. vectensis*, and *B. floridae* were remarkably similar to their human counterpart in terms of genomic exon/intron structure, protein size, and functional domains (Figures 4B and S4). No synteny analysis could be performed between starlet sea anemone and humans for *Nme5*, *Nme6*, and *Nme7* genes due to the limited number of genes present on corresponding sea anemone scaffolds (Table 2). Similarly to Nme7, no Nme8 ortholog was identified in *C. elegans*. In insects and *T. adhaerens*, the Nme8-related sequences that could be identified were extremely divergent (Figure 5A, Figure S2) and lacked the 3 NDPK_TX domains found in all eumetazoan Nme8 proteins, including the starlet sea anemone (Figure 5B). A conserved synteny was also identified between human and sea anemone (Figure 5C). Together our data suggest that, in contrast to *Nme5* and *Nme6* that have been identified in all investigated metazoan species, *Nme7* and *Nme8* have been lost in *C. elegans*. In insects, very divergent *Nme7* and *Nme8* genes remain. For *Nme8*, the high divergence associated with the loss of the 3 NDPK_TX domains in insects and *T. adhaerens* suggests a fast evolution of the gene possibly associated with a loss of ancestral metazoan Nme8 function.

We have shown that, in addition to the complexity of the Nme family, several highly conserved gene structures and protein domains are also conserved throughout metazoan evolution, some features being also conserved throughout the eukaryotic lineage. When considering the entire Nme family, the starlet sea anemone is the metazoan species exhibiting the most conserved gene and protein sequence features with humans.

### Starlet sea anemone as a model to investigate evolutionarily conserved *Nme* functions

Several non vertebrate model species, mainly *C. elegans* and *D. melanogaster*, have been used to investigate Nme functions. This fruitful approach has shed light on evolutionary conserved mechanisms involved in Alzheimer's disease [15], ciliary function [24], or epithelial integrity [25]. Nevertheless, the need for studies of Nme functions in non-vertebrate model species was recently stressed by the scientific community [16]. Here, we show that the starlet sea anemone exhibits a full set of metazoan Nme genes and shares remarkably conserved gene and protein sequence features with humans that were lost in flies and *C. elegans*. The starlet sea anemone is an emerging model [26] offering several biological features such as separate sexes, inducible spawning, flagellated sperm, and external fertilization. This model species thus offers significant opportunities to investigate Nme gene functions and thus shed new light on Nme functions that remain poorly understood [16].

Nme proteins of the group I are involved in a wide variety of cellular and physiological processes including tumor metastatic potential. Using a reciprocal BLASTP strategy, the starlet sea anemone genome was searched for homologs of human proteins known to interact with group I NME proteins. Among the 48 human proteins known to interact with NME1, NME2, NME3, or NME4, 44 had a homolog in sea anemone (Table S1). For instance, homologs of proteins involved in cancer and cell cycle control, such as MIF [27] and Rac1 [28], were clearly identified in sea anemone (Table S1). In addition, NME1 and NME2 have been demonstrated to regulate the expression of specific genes such as *c-MYC* [29], [30] and *p53* [31]. Interestingly, *c-MYC* and *p53* homologs could also be identified in the starlet sea anemone genome thus suggesting that at least some down-stream targets of Nme proteins are also present in this species (Table S1). Interestingly, the importance of p53 in sea anemone development was recently stressed and found to be similar to its known function in vertebrate development [32].

As previously documented, most Nme proteins of the group II, with the exception of Nme6, have been associated with ciliary functions. They have been shown to play critical roles in spermatogenesis [33]–[35], sperm motility [2], development [36], and human conditions associated with primary ciliary dyskinesia [37]. The existence of orthologs in non-metazoan species was however unsuspected with the exception of the report of Nme7 in *C. reinhardtii* and *T. thermophila* [24]. In the present study, we have demonstrated that *Nme5*, *Nme6*, *Nme7*, and *Nme8* genes were present in the metazoan ancestor, while *Nme5*, *Nme6*, and *Nme7* were most likely present in the eukaryote ancestor. To date, only 7 proteins are known to interact with NME proteins of the group II. We were however able to identify homologs for 6 of these interacting partners in starlet sea anemone (Table S1).

As indicated above, functional evidence exist demonstrating the importance of Nme gene for key biological processes, including development, cell proliferation, ciliary function, and cancer. We have shown here that the complexity of the Nme family predates metazoan radiation and that all Nme proteins display functional domains that have been conserved throughout evolution. Using the starlet sea anemone we were able to show that most proteins known to interact with human NME were also found in the eumetazoan ancestor. Together, these observations suggest a participation of Nme genes in key cellular functions that have been conserved throughout evolution. In this context, the starlet sea anemone that exhibits a full set of highly conserved Metazoan group II Nme genes and appropriate biological features - such as separate sex, flagellated sperm, and asymmetrical expression patterns during development -offers major opportunities to investigate Nme functions.

### Conclusion

In summary, we demonstrated that the complexity of the Nme gene family initially thought to be restricted to chordates was also shared by the Metazoan ancestor. We also provide evidence suggesting that the complexity of the family is mainly a eukaryotic innovation, with the exception of *Nme8* that is likely to be a choanoflagellate/metazoan innovation. Remarkably conserved gene structures, genomic linkage, and protein domains were identified among metazoans, some features being also conserved in eukaryotes. When considering the entire Nme family, the starlet sea anemone is the studied metazoan species exhibiting the most conserved gene and protein sequence features with humans. In addition, we were able to show that most of the proteins known to interact with human NME proteins were also found in the starlet sea anemone. Together, our observations further support the association of Nme genes with key cellular functions that have been conserved throughout metazoan evolution.

## Materials and Methods

### Sequence analysis

All Nme sequences were identified using the following genome assemblies: human (*Homo sapiens*, Assembly GRCh37), xenopus (*Xenopus tropicalis*, Assembly V.4.1), zebrafish (*Danio rerio*, Assembly ZV8), tunicate (*Ciona intestinalis*, Assembly V.2.0), florida lancelet (*Branchiostoma floridae*, Assembly V.2.0), purple sea urchin (*Strongylocentrotus purpuratus*, Assembly NCBI V.2.1), fruit fly (*Drosophila melanogaster*, Assembly BDGP5), yellow fever mosquito (*Aedes aegypti*, Assembly AaegL1), red flour beetle (*Tribolium castaneum*, Assembly Tcas 3.0), nematode (*Caenorhabditis elegans*, Assembly WS214), polychaete worm (*Capitella teleta*, Assembly V1.0), owl limpet (*Lottia gigantea*, Assembly V.1.0), starlet sea anemone (*Nematostella vectensis*, Assembly V.1.0), placozoan (*Trichoplax adhaerens*, Assembly Grell-BS-1999 V.1.0), marine choanoflagellate (*Monosiga brevicollis*, Assembly V1.0), fungi (*Saccharomyces cerevisiae*, Assembly EF 2; and *Ustilago maydis*, Assembly 1), amoebozoa (*Dictyostelium discoideum*, Assembly V.2.1), alveolate (*Tetrahymena thermophila, Assembly 1.1)*, green plants (*Micromonas pusilla*, Assembly V.2.0; and *Chlamydomonas reinhardtii*, Assembly V.4.0) and heterolobosean (*Naegleria gruberi*, Assembly V.1.0). A large number of sequences were obtained from the NCBI NR database using human or zebrafish protein sequences as a query. When more than one sequence was obtained, the RefSeq one was preferentially selected. When no RefSeq sequence was available, the longest sequence was used. When no sequences were available in the NR database, BLASTP was used on the Ensembl [38] and DoE Joint Genome Institute databases. The chromosomal localization of *Nme* genes was established using the Ensembl genome browser or JGI gene information, or when not available, using the UCSC Genome Bioinformatics BLAT [39] and the NCBI Sequence Viewer. Exon/intron structure was obtained from the Ensembl, NCBI, or JGI databases. The protein domain structure of Nme proteins was obtained from the GenBank Conserved Domain Database [40].

### Phylogenetic analysis of *Nme* proteins

Phylogenetic reconstructions were performed using the automated genomic annotation platform FIGENIX [41]. For each phylogenetic tree reconstruction, all selected protein sequences were added to a single multiple sequence alignment. Sequence alignment was performed automatically by the FIGENIX pipeline using MUSCLE v3.6 [42], [43]. The pipeline used is based on three different methods of phylogenetic tree reconstruction (i.e. neighbour-joining (NJ), maximum parsimony (MP), and maximum likelihood (ML)). The substitution model was calculated from data for ML while BLOSUM was used for NJ. Bootstrapping was carried out to assess node support with 1000 pseudoreplicates [44]. Support values were mapped onto a midpoint-rooted 50% majority rule consensus tree for each optimality criterion. Bootstrap values are reported for the nodes that are present in all three phylogenetic reconstruction methods. Asterisks denote the absence of a node for a given phylogenetic method.

### Synteny analysis

The synteny relationships of starlet sea anemone and human *Nme* genes were analyzed by reciprocal BLASTP on the NCBI NR database using surrounding genes of *Nme* genes in *Nematostella vectensis*. Homologous genes were considered in the analysis only when reciprocal BLASTP returned the couple as best hit. For the *Ciona intestinalis* paralogy analysis, synteny relationships were obtained using the Synteny Database [45] and putative paralogs were validated by reciprocal BLASTP on the NCBI NR database.

### Identification of *Nme* partners homologs

Validated human NME partners were obtained though NCBI Entrez Gene Interactions (http://www.ncbi.nlm.nih.gov/gene) information. Homologous genes in *Nematostella vectensis* were identified by reciprocal BLASTP on the NCBI NR database. Only BLASTP hits with an *E*-value lower than 10^−10^ were considered significant.

## Supporting Information

Figure S1 ***Ciona intestinalis*** **genomic region paralogy relationships between chromosomes 2q and 8q.** For *Ciona intestinalis* paralogy analysis, synteny relationships were inquired using the Synteny Database [45] and putative paralogs were validated by reciprocal BLASTP on NCBI NR databases. (TIF)

Figure S2 **Exon/intron structure of Nme** ***8*** **genes.** Exon/intron structure was obtained through Ensembl, NCBI, or JGI databases. When exon boundaries correspond to similar amino acid positions, the exons are displayed in color. Otherwise, exons are displayed in black. Non-coding exons are shown in grey. Numbers indicate exon size in nucleotides. (TIF)

Figure S3 **Phylogenetic reconstruction of the Nme protein family in eumetazoans.** Phylogenetic tree was constructed from a single multiple alignment. Bootstrap values for neighbor joining, maximum parsimony, and maximum likelihood methods, respectively, are indicated for each node. * indicates that the node does not exist in the corresponding tree. The consensus tree was calculated using the FIGENIX [41] automated phylogenomic annotation pipeline. Only *Homo sapiens*, *Danio rerio*, *Ciona intestinalis* and *Nematostella vectensis* sequences were used in this phylogenetic tree reconstruction because of the highly divergent ecdysozoans sequences greatly modifying the tree topology. (TIF)

Figure S4 **Exon/intron structure of Nme** ***7*** **genes.** Exon/intron structure was obtained through Ensembl, NCBI, or JGI databases. When exon boundaries correspond to similar amino acid positions, the exons are displayed in color. Otherwise, exons are displayed in black. Non-coding exons are shown in grey. Numbers indicate exon size in nucleotides. (TIF)

Table S1 **BLASTP hits of human NME partners against starlet sea anemone sequences.** Partners of human NME proteins were listed from NCBI Entrez Gene interaction information. BLASTP hits were considered significant for *e*-values lower than 10**^−^** ^10^. Accession numbers and BLASTP *e*-values are also given for c-Myc and p53, two downstream targets of human NME1 and NME2 proteins. (XLS)
