# Correction: Nme Gene Family Evolutionary History Reveals Pre-Metazoan Origins and High Conservation between Humans and the Sea Anemone, *Nematostella vectensis*

**DOI:** 10.1371/annotation/ad419d55-e0bd-4a2e-a79e-5265f44ae545

**Published:** 2010-11-17

**Authors:** Thomas Desvignes, Pierre Pontarotti, Julien Bobe

There was an error in affiliation 1 for authors Thomas Desvignes and Julien Bobe. Affiliation 1 should be: INRA, UR1037 SCRIBE, Campus de Beaulieu, F-35000 Rennes, France.

