# Supplementary figures and images for: Nme Gene Family Evolutionary History Reveals Pre-Metazoan Origins and High Conservation between Humans and the Sea Anemone, Nematostella vectensis
Source: PLoS One. 2010 Nov 11;5(11):e15506. doi: 10.1371/journal.pone.0015506 (PMC2978717; doi:10.1371/journal.pone.0015506)

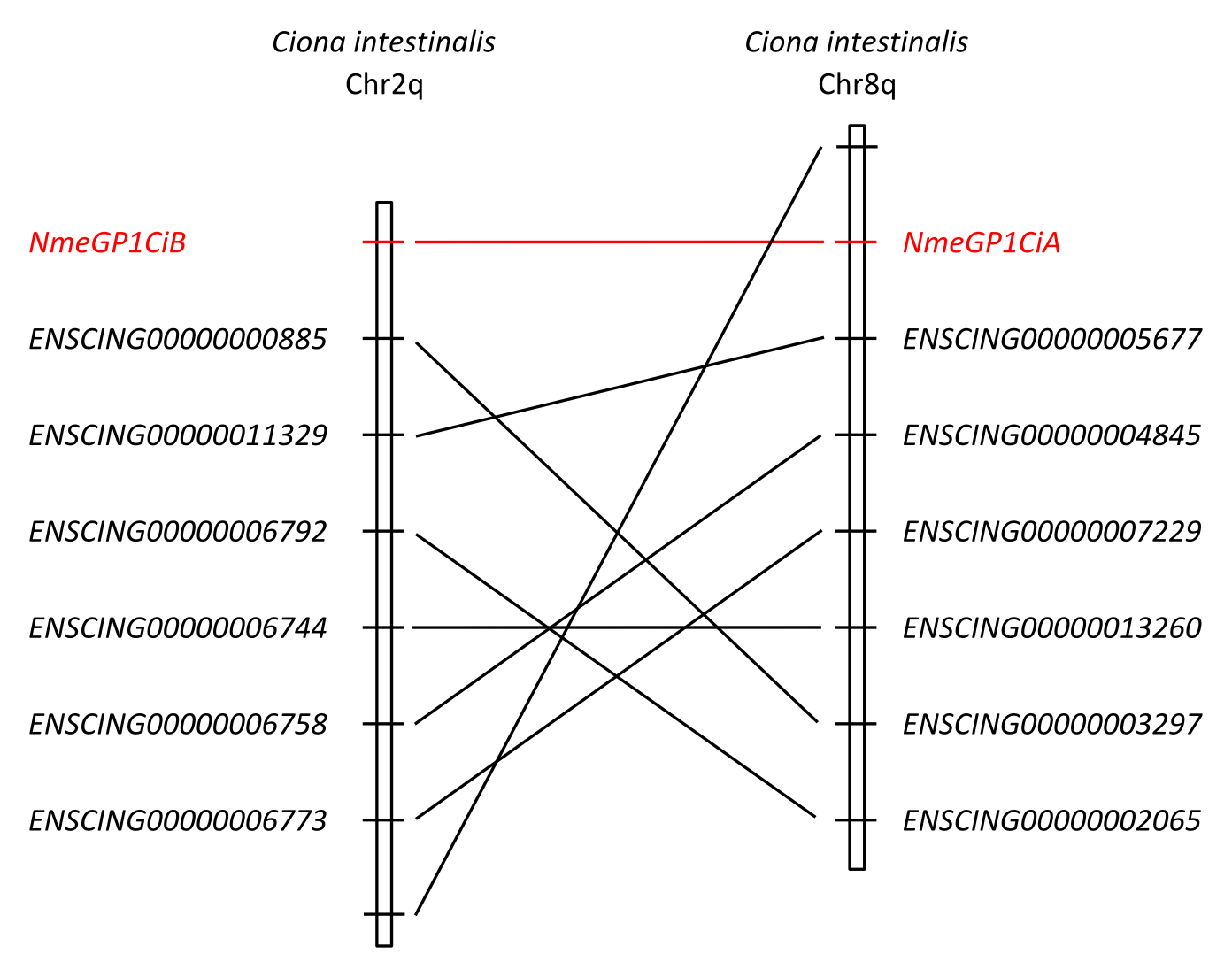

Supplement: Figure S1 — Ciona intestinalis genomic region paralogy relationships between chromosomes 2q and 8q. For Ciona intestinalis paralogy analysis, synteny relationships were inquired using the Synteny Database [45] and putative paralogs were validated by reciprocal BLASTP on NCBI NR databases. (TIF) [file pone.0015506.s001.tif]

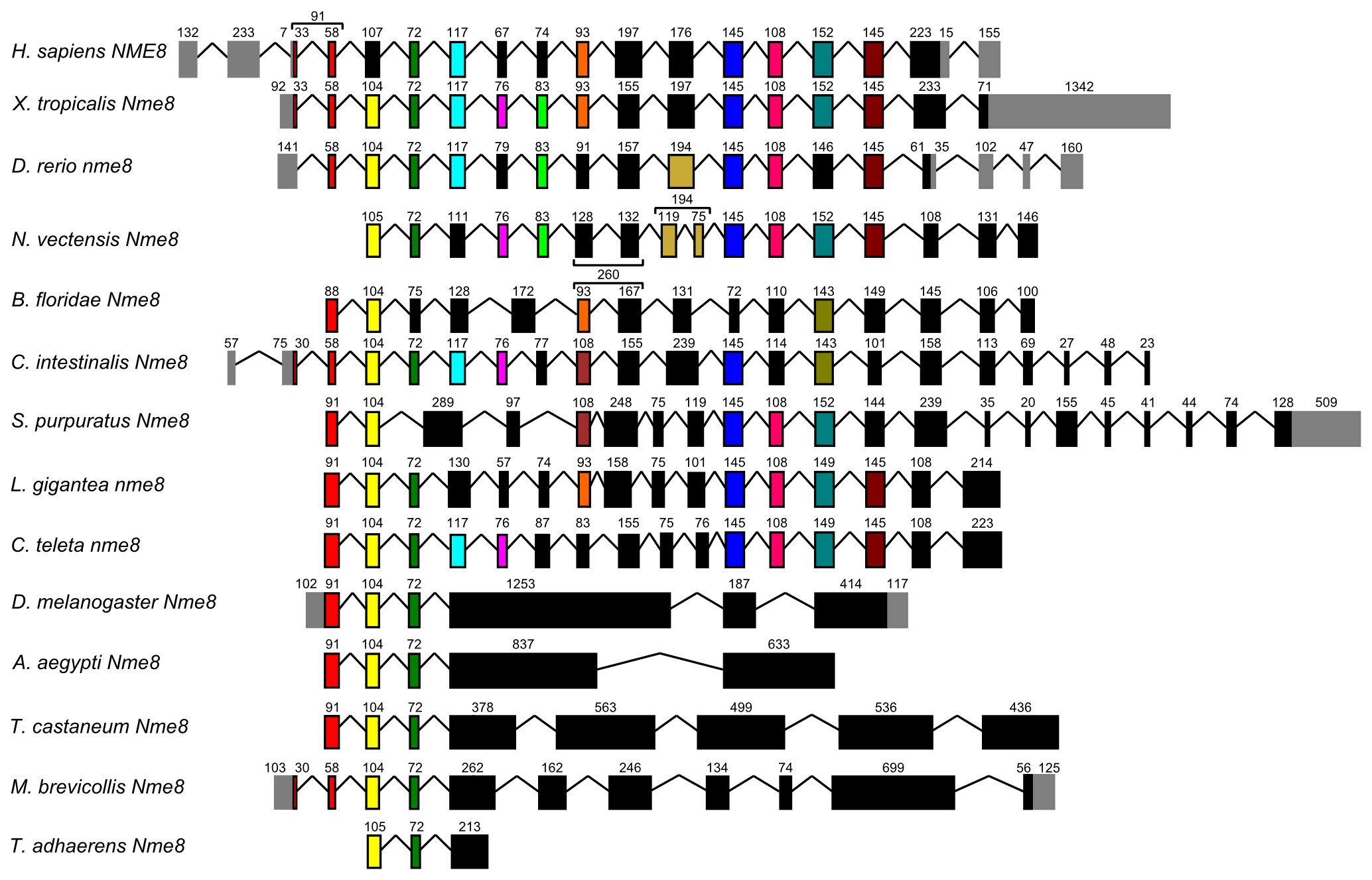

Supplement: Figure S2 — Exon/intron structure of Nme 8 genes. Exon/intron structure was obtained through Ensembl, NCBI, or JGI databases. When exon boundaries correspond to similar amino acid positions, the exons are displayed in color. Otherwise, exons are displayed in black. Non-coding exons are shown in grey. Numbers indicate exon size in nucleotides. (TIF) [file pone.0015506.s002.tif]

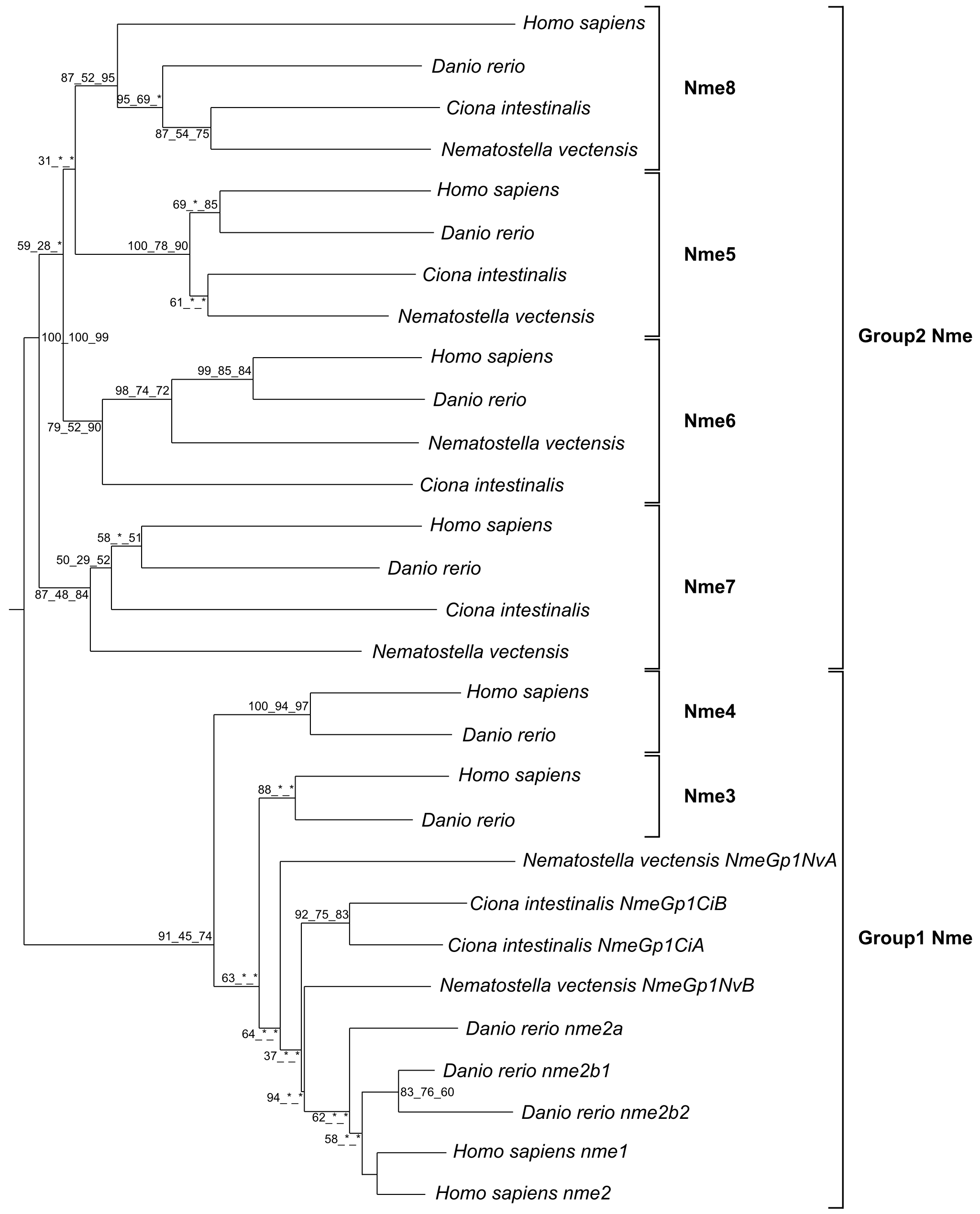

Supplement: Figure S3 — Phylogenetic reconstruction of the Nme protein family in eumetazoans. Phylogenetic tree was constructed from a single multiple alignment. Bootstrap values for neighbor joining, maximum parsimony, and maximum likelihood methods, respectively, are indicated for each node. * indicates that the node does not exist in the corresponding tree. The consensus tree was calculated using the FIGENIX [41] automated phylogenomic annotation pipeline. Only Homo sapiens, Danio rerio, Ciona intestinalis and Nematostella vectensis sequences were used in this phylogenetic tree reconstruction because of the highly divergent ecdysozoans sequences greatly modifying the tree topology. (TIF) [file pone.0015506.s003.tif]

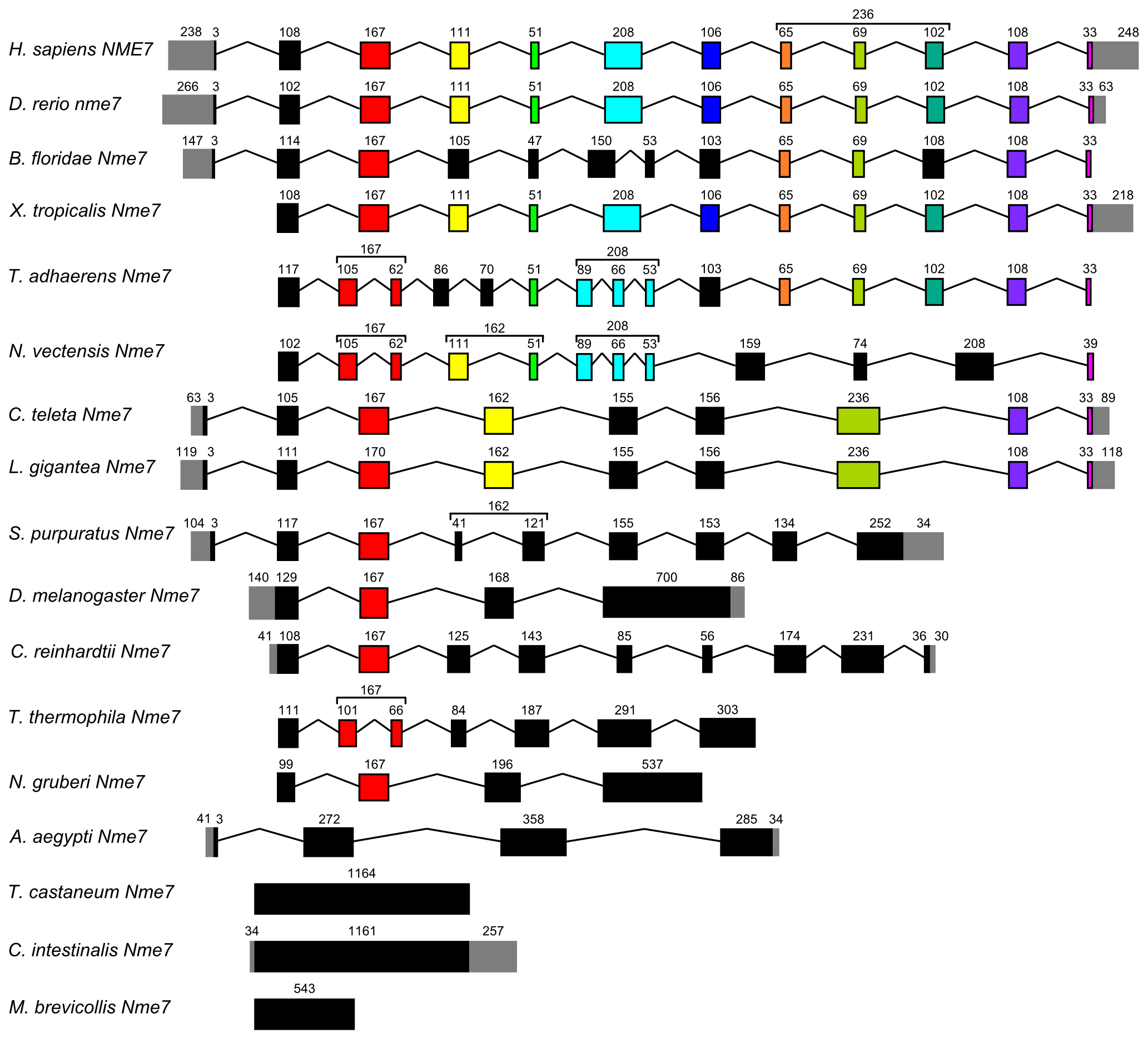

Supplement: Figure S4 — Exon/intron structure of Nme 7 genes. Exon/intron structure was obtained through Ensembl, NCBI, or JGI databases. When exon boundaries correspond to similar amino acid positions, the exons are displayed in color. Otherwise, exons are displayed in black. Non-coding exons are shown in grey. Numbers indicate exon size in nucleotides. (TIF) [file pone.0015506.s004.tif]
